# Genetic Alterations of Metastatic Colorectal Cancer

**DOI:** 10.3390/biomedicines8100414

**Published:** 2020-10-13

**Authors:** Ugo Testa, Germana Castelli, Elvira Pelosi

**Affiliations:** Department of Oncology, Istituto Superiore di Sanità, Viale Regina Elena 299, 00161 Rome, Italy; germana.castelli@iss.it (G.C.); elvira.pelosi@iss.it (E.P.)

**Keywords:** colorectal cancer, genomic alterations, metastasis, tumor heterogeneity, tumor evolution

## Abstract

Genome sequencing studies have characterized the genetic alterations of different tumor types, highlighting the diversity of the molecular processes driving tumor development. Comprehensive sequencing studies have defined molecular subtypes of colorectal cancers (CRCs) through the identification of genetic events associated with microsatellite stability (MSS), microsatellite-instability-high (MSI-H), and hypermutation. Most of these studies characterized primary tumors. Only recent studies have addressed the characterization of the genetic and clinical heterogeneity of metastatic CRC. Metastatic CRC genomes were found to be not fundamentally different from primary CRCs in terms of the mutational landscape or of genes that drive tumorigenesis, and a genomic heterogeneity associated with tumor location of primary tumors helps to define different clinical behaviors of metastatic CRCs. Although CRC metastatic spreading was traditionally seen as a late-occurring event, growing evidence suggests that this process can begin early during tumor development and the clonal architecture of these tumors is consistently influenced by cancer treatment. Although the survival rate of patients with metastatic CRC patients improved in the last years, the response to current treatments and prognosis of many of these patients remain still poor, indicating the need to discover new improvements for therapeutic vulnerabilities and to formulate a rational prospective of personalized therapies.

## 1. Introduction

Colorectal cancer (CRC) is one of the most frequent cancers worldwide, corresponding to the second in males and third in females most frequent tumor. CRC is the second most common cause of cancer death in Europe [1].

Colorectal cancer is a highly heterogeneous disease that comprises different tumor phenotypes, characterized by specific molecular and morphological alterations. CRC is caused by genetic alterations that target tumor suppressor genes, oncogenes, and genes related to DNA repair mechanisms. Depending on the origin of these mutations, CRC can be classified as sporadic (70–75%), hereditary (5%), and familial (20–25%). Three major pathways are involved in CRC origin and progression: (a) chromosomal instability (CIN); (b) microsatellite instability (MSI); (c) CpG island methylation phenotype (CIMP). Each of these three different groups displays peculiar pathological, genetic, and clinical characteristics [2].

CIN is the most common (85% of total CRCs) genetic mechanism occurring in CRC. CIN is characterized by the acquisition of a consistent karyotypic variability, aneuploidy, chromosomal and subchromosomal aberrations, gene amplifications and loss of heterozygosity. Allelic losses at the level of chromosome arms 1p, 5q, 17p, 18p, 18q, 20p, and 22q are highly recurrent. A major pathogenic consequence of this CIN consists in the loss of heterozygosity at tumor suppressor gene loci. Furthermore, CIN tumors are associated with the accumulation of mutations at the level of several oncogenes, including *KRAS* and *BRAF* and of tumor suppressor genes such as *APC* and *TP53*. The meta-analysis of the outcome of more than 10,000 CRC patients clearly indicated that CIN is associated with a worse prognosis [3].

MSI involves several recurrent alterations in the microsatellite zone, without apparent structural and numerical changes in the genome; approximately 15% of all CRCs have a high frequency of MSI due to germline mutations in mismatch repair (MMR) system or somatic inactivation by promoter hypermethylation of MLH1 gene [4].

CIMP pathway is responsible for 20–30% of total CRCs and is predominantly observed in the proximal colon (30–40%) and more rarely in distal colon (3–12%) [4].

The Cancer Genome Atlas provided in 2002 the first genome-scale analysis of a large set (276) of CRC samples, performing a comprehensive study involving exome sequencing, DNA copy number, promoter methylation, messenger RNA and micro RNA expression evaluation [4]. This analysis showed that CRCs can be classified according to their mutation pattern: (i) 16% of CRCs were found to be hypermutated (75% displayed high MSI, usually associated with hypermethylation and silencing of the *MLH1* gene, whereas the remaining 25% exhibited mismatch-repair gene and polymerase ε (POLE) gene mutations); (ii) the non-hypermutated CRCs that formed the most consistent group of tumors showed the recurrent mutations of *APC*, *TP53*, *KRAS*, *PIK3CA*, *FBXW7*, *SMAD4*, *TCF7L2*, and *NRAS* genes; (iii) in hypermutated CRCs, the most frequently mutated genes were *ACVR2A* (63%), *APC* (51%), *TGFBR2* (51%), *BRAF* (49%), *MSH3* (46%), *MSH6* (40%), *MYO18* (31%), *TCF7L2* (31%), and *CASP8* (29%); (iv) *APC* (81% vs. 51%) and *TP53* (60% vs. 20%) were significantly more mutated in the non-hypermutated cancers compared to hypermutated cancers. Integrated analysis of the genetic profiling showed that some pathways are recurrently altered in CRCs: (i) WNT pathway is altered in 93% of all tumors (in 80% of cases due to biallelic inactivation of *APC* or activating mutations of *CTNNB1*); (ii) PI3K signaling pathway is altered in 50% of non-hypermutated and 53% of hypermutated CRCs; (iii) RTK-RAS signaling pathway is more frequently altered in hypermutated (80%) than in non-hypermutated (59%); (iv) finally, TGF-β signaling pathway was much more frequently altered in hypermutated (87%) than in non-hypermutated (27%) CRCs [4].

The study by TCGA showed the existence of three subtypes of CRC according to their transcriptomic profile: microsatellite instability/CpG island methylator phenotype (MSI/CIMP); invasive; chromosome instability (CIN). In a subsequent study, Zhang et al. carried out a proteogenomic analysis on the CRCs previously characterized by TCGA [5]. This analysis showed the existence of a limited correlation between mRNA and protein levels. Five CRC subtypes (from A to E) were identified according to proteomic data: (i) B and C subtypes included all CRCs characterized by hypermutation, MSI-H, *POLE* and *BRAF* mutations: B subtype was associated with the CIMP-H methylation subtype of the TCGA study, absence of *TP53* mutations and chromosome 18 loss; C subtype was associated with a non-CIMP TCGA subtype. (ii) The A, D, and E subtypes were associated with the TCGA CIN subtype. (iii) The E subtype displayed several remarkable features, such as the presence of *TP53* mutations and chromosome 18q loss (both genomic alterations frequently associated with CIN CRCs) and with *HNF4A* amplification and HNF4α protein abundance [5]. CRCs display frequent copy number alterations (CNAs), particularly those characterized by CIN. However, only few CNAs are associated with significant changes at protein level. Among the various CNAs, the chromosome 20 amplicon was associated with the largest changes at both mRNA and protein level and is associated with HNF4 (hepatocyte nuclear factor 4, alpha), TOMM34 (translocase of outer mitochondrial membrane 34) and SRC (SRC proto-oncogene, non-receptor tyrosine kinase) overexpression [5].

Copy number alterations (CNAs) show significant changes during the progression of colorectal carcinogenesis from benign adenoma to CRC. Thus, chromosomal aneuploidies affecting chromosomes 7, 13, and 20q (all chromosomal gains) cooperate with APC mutations in the progression from adenoma with low-grade dysplasia to adenoma with high-grade dysplasia. Losses of chromosomes 8p, 15q, 17p, and 18q and gain of 8q are involved in tumor progression to infiltrating adenocarcinoma [6].

The analysis of gene expression profiles obtained through the study of thousands cases of colorectal cancers supported a classification of colon cancer, based on four major consensus molecular subtypes (CMS), CMS1 to CMS4 (Table 1) [7]. CMS1 group (MSI immune subtype, including 14% of all CRCs) is characterized at genetic level by hypermutation, hypermethylation, enrichment for *BRAF^V600E^* mutations (observed in 40% of these tumors) and by pronounced infiltration of the tumor microevironment by immune cells, particularly represented by T lymphocytes (both Cytotoxic CD8^+^ and CD4^+^ T helper) and natural killer lymphocytes; frequent in these tumors are mutations at the level of *APC* (35%), *TP53* (30%) and *KRAS* (25%) genes. Frequent in these tumors are mutations in *MSH6*, *RNF43*, *ATM*, *TGFBR2*, *BRAF*, and *PTEN* genes. Predominantly, these tumors originate from precursor lesions with a serrated histology, with preferential location at the level of proximal regions of the colon; their prognostic outcome is intermediate but poor after relapse. The CMS2 subtype corresponds to the canonical subtype (37% of CRCs) and is characterized by CIN-high, microsatellite stability (MSS) and low levels of gene hypermethylation; a mutational profile typically observed in CIN-high CRCs, including recurrent *APC* (75%), *TP53* (70%), and *KRAS* (30%) mutations, whereas *BRAF* mutations were absent; pronounced upregulation of WNT and MYC downstream targets, elevated expression of EGFR, HER2, IGF2, IRS2, HNF4A, and cyclin; complex tubular histological structure, predominantly located in the distal region of the colon. The CMS3 subtype corresponds to the metabolic subtype (10% of CRCs) that is characterized by activation of glutaminolysis and lipidogenesis and by the presence of a distinctive genomic and epigenomic profile compared with other CIN tumors, for the presence of a mixed CIMP-H (20% of cases), MSI-H (15% of cases), hypermutation (30% of cases), and CIN-H (54% of cases); at mutational level, frequent *KRAS* and *APC* mutations but less frequent *TP53* and *BRAF* mutations are observed; these tumors predominantly display papillary morphology and are located at the level of both proximal and distal regions of colon. CMS4 corresponds to the mesenchymal subtype (25% of all cases) and is characterized by the presence of tumors exhibiting activation of the pathways related to epithelial-mesenchymal transition (EMT) and stemness (TGF-β signaling and integrins) and overexpression of genes involved in extracellular matrix remodeling, complement-associated inflammation, stromal invasion and angiogenesis; marked stromal cell infiltration at the level of peritumoral microenvironment is a typical histological feature of these tumors; these tumors are frequently CIN-H but rarely hypermutated, CIMP-H and MSI-H; at mutational level, frequent are the mutations of *APC*, *TP53* and *KRAS*, associated with rare *BRAF* mutations; at histological level, these tumors are characterized by a desmoplastic reaction with high stroma; these tumors are associated with a poor outcome compared with the other CMS subtypes [7].

Finally, there is a residual unclassified group representing 10–15% of all tumors with mixed features, that seemingly represents a transitional phenotype or reflects an intra-tumoral heterogeneity [7].

Importantly, the CMS classification was predictive of chemotherapy and targeted-therapy response in CRC patients with advanced/metastatic disease [8,9,10].

The CMS transcriptional classification was implemented through the analysis of microenvironment signatures, showing consistent correlation between these two classification systems: CMS1 subgroup was characterized by elevated expression of genes specific to cytotoxic T lymphocytes; CMS4 subgroup was characterized by several microenvironmental features, including expression of monocytic markers and a combined angiogenesis, inflammatory and immunosuppressive signature; at pathologic level, CMS4 tumors display numerous infiltrating fibroblasts, producing cytokines and chemokines inducing the angiogenetic and inflammatory phenotypes; CMS2 and CMS3 subgroups exhibit low inflammatory and immune signatures [11].

Isella and coworkers have proposed a new transcriptional classification of CRC, allowing the identification of five CRC intrinsic (CRIS) subtypes, displaying distinctive molecular, phenotypic and functional features [12]. This classification was based on a methodological approach to limit the impact of tumor stromal cells on the transcriptional classification of CRC. CRIS-A identifies a subgroup of CRCs enriched for MSI-H, *BRAF* or *KRAS*-mutated tumors, with secretory mucinous histology, with sustained glycolytic metabolism and inflammatory traits; CRIS-A englobes CRCs mainly corresponding to CMS1 and, at a minor extent, CMS4. CRIS-B identifies a subset of CRCs characterized by an impaired differentiation, activation of TGF-β signaling and epithelial to mesenchymal transition; these tumors are mainly MSS and only in part MSI-H; these tumors are characterized by a poor prognosis and by an elevated infiltration of fibroblasts; CRIS-B englobes both CMS4 and CMS1 tumors. CRIS-C identifies a group of CRCs, CIN-H, and MSS, with absent *KRAS* mutations and exhibiting elevated EGFR activity and *MYC* copy number gains; these tumors are particularly sensitive to EGFR inhibitors; CRIS-C englobes CMS2 tumors and in part CMS4 tumors. CRIS-D tumors display a number of typical features mainly represented by a stem-like phenotype associated with high WNT signaling, a MSS status, strong enrichment of IGF2 overexpression/amplification and FGFR autocrine stimulation; CRIS-D englobes both CMS2 and CMS4 tumors. CRIS-E is characterized by a Paneth cell-like phenotype, an MSS status, numerous WNT-related features, and frequent *TP53* mutations; CRIS-E englobes both CMS2 and CMS4 CRCs [12].

The complex, variable and potentially confounding role of microenvironment in the evaluation of the transcriptomic expression of CRC, highlights the need of performing analyses at single-cell level as a tool to better define and understand intratumoral heterogeneity [13]. Only few studies have explored single-cell transcriptomic in CRC samples. In this context, particularly relevant was the study carried out by Li and coworkers investigating single-cell RNA sequencing on 969 tumor cells derived from primary tumors of 11 different CRC patients and 622 normal mucosal intestinal cells located near the CRC [14]. This analysis identified seven different cell clusters, corresponding to epithelial cells, endothelial cells, fibroblasts, T lymphocytes, B lymphocytes, mast cells, and myeloid cells [14]. The single-cell analysis allowed the identification of a larger set of differentially expressed genes compared with normal mucosa than the bulk analysis of gene expression [14]. Importantly, EMT (epithelial-mesenchymal transition)-related genes resulted to be upregulated only at the level of the cell population of cancer-associated fibroblasts but not at the level of epithelial cells [14]. The data obtained from single-cell transcriptomic allowed to define six different signatures of six tumor cell types: epithelial differentiated; epithelial stem, fibroblast, T cell, B cell, macrophages [14]. The integration of the six cell type signatures together with the data of bulk signatures obtained through the analysis of various cohorts of CRC patients allowed to define three tumor groups, defined as S1, S2 and S3: S1 CRCs display a weak epithelial, an elevated myeloid and a strong fibroblast signature; S2 CRCs exhibit intermediate level of all signatures; S3 CRCs show a strong epithelial signature, associated with weak myeloid and fibroblast signatures [14]. In all the cohorts of CRC patients studied, S3 CRCs display a better survival than the two other groups [14]. A more recent study based on the analysis of >50,000 single cells from CRCs and matched normal tissues provided evidence that CRC development is associated in all cases analyzed with changes at the level of epithelial, immune and stromal cell compartments [15]. Interestingly, in the epithelium, five different tumor-specific stem and progenitor-like cell populations were identified [15]. This single-cell analysis showed also that epithelial tumor cells and cancer-associated fibroblasts are fundamental and essential for the assignment of each CRC to a given CMS subtype [15].

Although single-cell transcriptomic techniques cannot be proposed for the clinical classification of CRCs, their use may be of considerable support in the study of CRC patients undergoing immunotherapy treatments or myeloid-targeted therapies [16,17].

Very few studies have explored the gene expression profile observed at the level of metastatic CRC lesions. Kamal et al. reported the comparative analysis of the transcriptomic profile of primary tumors and corresponding metastases (liver and lung metastases) in some CRC patients [13]. According to the gene expression profile, two types of distant metastases were identified: M1 and M2 [13]. The M1 metastatic group is characterized by strong activation of inflammatory and immune response pathways (including immune evasion pathways, such as those involving PD-1/-L1 signaling) and enrichment in EMT activity. The M2 metastatic group exhibits MYC activation and cell proliferation [18]. Importantly, treatment modifies the gene expression profile of metastatic lesions: the immune phenotype of M1 metastases is lost in post-treatment metastases; treatment induces an enrichment of EMT activity [18]. The analysis of CMS groups in metastases showed the absence of CMS3 and the presence of CMS1 in only few cases; the majority of metastases were classified as CMS2 (37%) or CMS4 (45%); 86% of metastases were CMS4 in the M1 cluster, while 60% of metastases were CMS2 in the M2 cluster [18]. The comparison of gene expression in paired primary tumors and corresponding metastases showed that *FBN2* and *MMP3* were the most differentially expressed genes [18].

The incidence of CRC increases with the age. In a recent study, Lieu et al. on a large panel of CRC samples reported the occurrence of CRCs in 7.8% of patients under the age of 40, 17.6% in the age comprised between 40 and 49 years and 74.6% in patients with an age of 50 or older [19]. Overall genomic alterations were similar in the majority of genes currently mutated, with some notable differences: in MSS CRC patients, *TP53* and *CTNNB1* alterations were more common in younger patients with CRC [19]; in the MSI-H cohort, most of genes displayed a similar frequency of alterations in the two age groups, but significant differences were observed at the level of *APC* and *KRAS* alterations more frequent among younger than older patients and *BRAF* alterations markedly more recurrent among older than younger CRC patients [19].

The progresses made in primary and adjuvant treatments of CRC patients have led to an improvement of the survival times of these patients. The optimal treatment of CRC patients would imply complete surgical ablation of primary tumor and metastases. However, 25–30% of CRC patients display at diagnosis an advanced disease stage with metastatic diffusion; furthermore, a remaining 20% of patients develop metachronous metastases after standard treatments. Therefore, a significant proportion of CRC patients need an efficacious medical treatment to induce the regression of tumor cells that cannot be removed by surgery. The current medical treatment implies first line chemotherapy or radiotherapy that can be performed either before surgery in a neoadjuvant setting or after surgery in an adjuvant setting. Current chemotherapy treatment implies either single-drug treatment involving fluoropyrimidine (5-FU) and multiple-drug regimens, based on the use of irinotecan (IRI), capecitabine (CAP) or oxaliplatin (OX), such as FOLFOX (5-FU + OX), FOXFIRI (5-FU + IRI), CAPIRI (CAP + IRI) or CAPOX (CAP + OX) [1].

The studies carried out in the last years have shown that CRC exhibits a clinically relevant molecular heterogeneity related to various genetic and non-genetic mechanisms. The identification of molecular subtypes of CRCs helped to identify new strategies of treatment for selected groups of patients (targeted therapy): (i) the presence of *KRAS* or *NRAS* mutations allowed the identification of a group of CRC patients refractory to EGFR inhibitors; (ii) the absence of *KRAS, NRAS, BRAF*, and *PIK3CA/PTEN* mutations (CRC “wild-type”) identifies a group of CRC patients responsive to EGFR inhibitors; (iii) CRCs bearing *BRAF^V600E^* mutations have a poor prognosis and are responsive to targeted inhibition in combination; (iv) CRCs with *HER2* amplifications display sensitivity to dual HER2 blockade; (v) CRCs bearing rare kinase fusion events are targetable with specific kinase inhibitors; (vi) MSI-H and *POLE* hypermutant CRCs are particularly sensitive to treatment with immune checkpoint inhibitors; (vii) CRCs with a mesenchymal phenotype display immunosuppressive mechanisms that could be removed through combined immunotherapy treatments [20].

The strategy recommended by the National Comprehensive Cancer Network (NCCN) for the targeted therapy of metastatic CRC patients implies a differential treatment according to the *RAS* mutational status and to the colon location of the primary tumor: (i) for patients with left colon mCRC, *RAS*-WT it is recommended an initial therapy based on EGFR inhibitors, and a subsequent therapy based on mutational status for *BRAF* mutations (BRAF inhibitors), *HER2* amplifications (HER2 inhibitors) *BRAF/HER2*-WT (anti-PD-1/L1 if deficient in mismatch repair (MMR); anti-VEGF if proficient in MMR); for patients with right colon mCRC it is recommended a therapeutic approach similar to that adopted for *RAS*-mutant patients; for patients with mCRC, RAS-mutant it is recommended a differential therapy according to the MMR status: for patients deficient in MMR it is recommended a first-line of therapy based on anti-PD-1/L1 and a second line based on anti-VEGF inhibitors, whereas for patients proficient in MMR, a first line based on anti-VEGF inhibitors and a second line of therapy based on best supportive care therapy are recommended [21].

A large body of molecular data on the genomic abnormalities observed in CRC has been generated; the majority of these studies focused on primary tumors. However, recent studies have characterized the molecular abnormalities observed in metastatic CRC. Some studies have molecularly characterized metastatic lesions with their corresponding primaries. The present review paper reports a detailed analysis of these recent studies on the characterization of metastatic CRCs, supporting the view that a better understanding of the molecular alterations and of their heterogeneity may improve the treatment outcome of these patients.

## 2. Genetic Abnormalities in Metastatic CRC

Few studies have explored the frequency of recurrent genetic alterations in metastatic CRC patients.

In 2017, Zehir and coworkers reported the mutational landscape of 10,945 metastatic tumors, including 975 metastatic CRCs, as encountered in clinical practice [22]. This study showed the presence of four recurrently mutated genes, represented by *APC*, *TP53*, *KRAS*, and *PIK3CA*. Furthermore, according to the somatic tumor burden, metastatic CRCs can be distinguished into three groups: normal, hypermutated, ultramutated. The metastatic CRCs with a high mutational burden displayed a dominant MMR signature. Finally, 35% of metastatic CRCs showed actionable somatic alterations [22].

**Figure 1:**
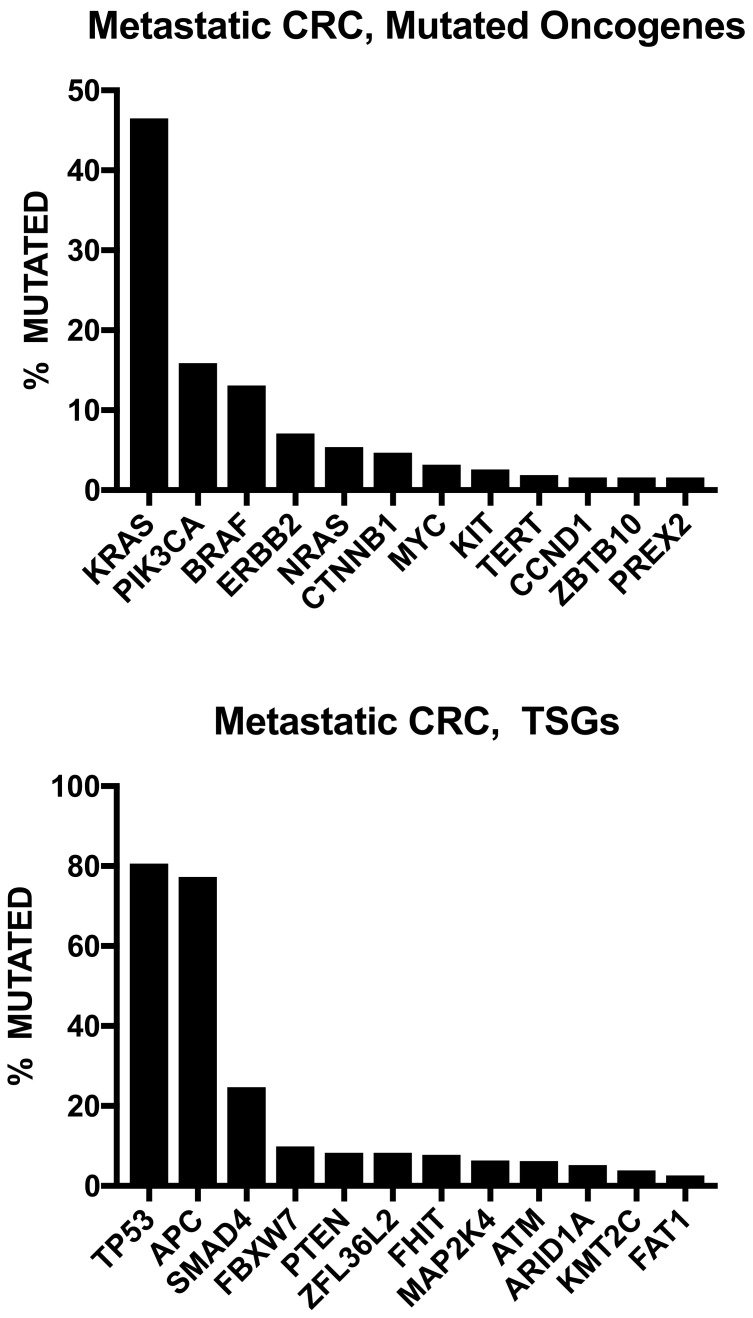
Frequency of the most recurrent gene alterations observed in metastatic CRC patients. The data on the frequency of the major genetic alterations were reported by Priestly et al. [23] and were based on the wide-genome sequencing analysis of 372 metastatic CRC patients.

The study carried out by Zehir et al. was based on targeted gene analysis [22]. A more recent study by Priestley et al. involved deep whole-genome sequencing of 2399 metastatic solid tumors, including 372 CRCs [23]. Metastatic CRCs are among the tumors displaying the highest levels of single-nucleotide variants (SNVs), with only urinary tract, esophagus, lung cancers and melanoma exhibiting higher levels among 20 different types of metastatic cancers [23]. Only 4% of metastatic CRCs displayed an MSI genotype/phenotype, a frequency that is lower than that reported for primary CRC, a finding that can be explained by the lower tendency of these tumors to metastasize [13]. Copy number alterations are frequent in metastatic CRC; an extreme form of CNA can be caused by whole genome duplication (WGD), an event frequent (>60% of cases) in metastatic CRCs, among the metastatic tumors most frequently showing WGD [23]. Metastatic CRCs displayed a mean number of total candidate driver events (6.5 per patient) only slightly higher than the mean number (5.7 per patient) observed in 20 different metastatic cancers [23]. The whole-genome sequencing (WGS) approach allowed to accurately define the frequency of genetic alterations occurring in mCRC at the level of genes possessing oncogenic activity when mutated or of tumor suppressor genes (Figure 1). The analysis of the co-mutation pattern of driver genes showed negative associations within the same transduction pathway for *KRAS-BRAF* and *KRAS-NRAS*, for *APC-CTNNB1*, for *APC* with *BRAF* and *RNF43* [23]. Interestingly, this study showed in 9 CRC patients with absent *APC* driver mutations, the occurrence of in-frame deletion of the complete exon 3, leading to activation of the WNT and β-catenin pathway [23]. Furthermore, 5.4% of mCRC samples displayed an amplification of *CDX2*, acting as a survival oncogene for these tumor cells [23]. The exploration of the mutational spectrum of metastatic CRC indicates that only 30% of these tumors possess biomarkers with either an approved therapy or with strong biological evidence or clinical trials that are actionable [23].

**Figure 2:**
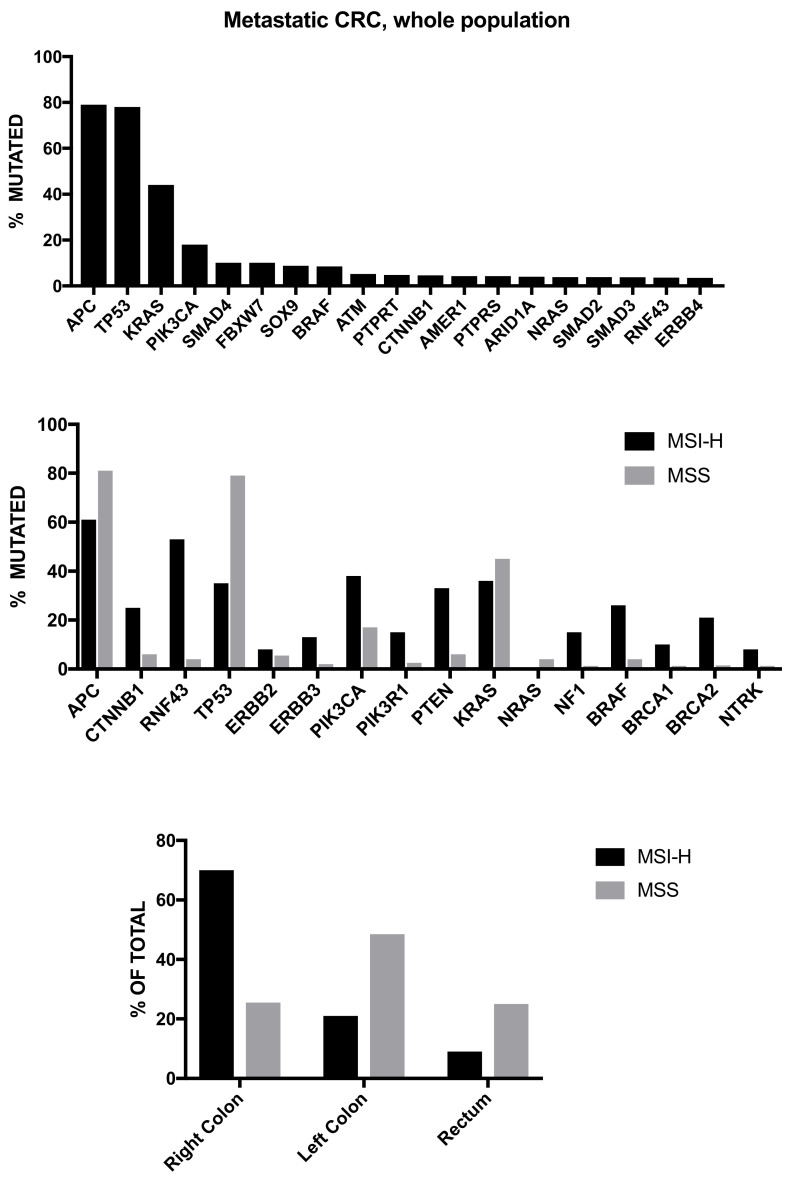
Frequency of the most recurrent genetic alterations observed in metastatic CRC patients (data reported by Yaeger et al., 2018) [24]. Top Panel: most recurrent genetic alterations observed in the whole population of metastatic CRC patients; Middle Panel: most recurrent genetic alterations observed in the population of metastatic CRC patients subdivided into MSI-H and MSS; Bottom Panel: tumor location in metastatic CRC patients exhibiting either MSI-H or MSS.

Particularly relevant was the study carried out by Yaeger et al. [24] who reported the sequencing analysis of most 1134 CRCs, including 979 patients with metastatic disease. These tumors corresponded to three different molecular groups: POLE mutant (0.7%), MSI-H/hypermutated (8.7%) and MSS (90.5%), with predominant left colon localization of MSS tumors and predominant right colon localization of POLE and MSI-H tumors [24]. The WNT pathway resulted to be altered in 85% of MSS tumors and in 93% of MSI-H tumors: *APC* gene alterations were more frequent in MSS CRCs than in MSI-H CRCs (81% vs. 61%), while *CTNNB1* and *RNF43* gene alterations were less frequent in MSS CRCs than in MSI-H CRCs (6% vs. 25% and 4% vs. 53%, respectively) [24] (Figure 2). Other remarkable differences in the rates of several genetic alterations between these two types of metastatic CRCs are represented by the more frequent alterations of *ERBB3*, *PIK3CA*, *PIK3R1*, *PTEN*, *NF1*, *BRAF*, *BRCA1*, and *BRCA2* gene alterations in MSI-H CRCs than in MSS CRCs [24]. (Figure 2) The analysis of mostly recurrently mutated genes in MSS CRCs showed a mutational frequency of 79% for *APC*, 78% for *TP53*, 44% for *KRAS*, 18% for *PIK3CA*, 16% for *SMAD4*, 10% for *TCF7L2* and 10% for *FBXW7* [24]. The analysis of the frequencies of some gene mutations in early-stage tumors, primary metastatic CRC and metastases from metastatic CRCs showed that most of these mutations do not display significant differences, but a minority of them are stage-related: the frequency of *TP53* mutations progressively increases from early-stage to primary mCRC and to metastases of mCRC; *FBXW7* mutations are more frequent in early-stage and primary mCRCs than in metastases of mCRC; *ERBB2* mutations are more frequent in early-stage than in metastatic CRCs [24]. *BRAF* mutations display a tendency to be more frequent in metastatic CRC than in early-stage CRC [24]. This study also showed some remarkable differences between primary tumor sites, i.e., right colon or left colon. Right-sided primary mCRC displayed fewer DNA copy-number alterations than left-sided mCRC; furthermore, an enrichment of genetic alterations in *KRAS*, *BRAF*, *PIK3CA*, *PTEN*, *AKT1*, *RNF43*, *SMAD2*, and *SMAD4* was observed in right-sided primary mCRC and in *APC* and *TP53* in left-sided primary mCRC [24]. Left-located mCRC had a significantly better overall survival than right-located mCRC [24]. The analysis of the overall survival in various molecular subgroups of mCRCs showed a poor survival for patients bearing *KRAS* mutations alone or in combination with PI3K pathway mutations. These CRCs showed also a greater tendency to have multiple first sites of metastases [24].

Using a multigene panel sequencing, Belardinilli and coworkers have explored the co-mutational profile of metastatic CRC; this study involved the analysis of 779 metastatic CRC primary tumors [25]. The results of this analysis showed the existence of positive associations between *EGFR* and *KRAS*, *EGFR* and *SMAD4*, *BRAF* and *PTEN*, and *NRAS* and *TP53* mutations, whose biological and clinical significance is at the moment unknown [25]. Importantly, according to the presence of *TP53* and *KRAS* mutations, metastatic CRCs can be subdivided into four different groups: MAP1, characterized by the co-mutation of *TP53* and *KRAS* and subdivided into a less frequent MAP1.1 subgroup, in which *TP53* and *KRAS* mutations are associated with other recurrent mutations, such as *PIK3CA*, *FBXW7*, *SMAD4* and *PTEN* mutations and a more frequent MAP 1.2 subgroup in which *TP53* and *KRAS* mutations are not associated with other recurrent mutations; MAP 2, characterized by the mutation of the *KRAS* gene and subdivided into a MAP 2.1 subgroup in which *KRAS* mutation is associated with highly recurrent *PIK3CA* mutations and a MAP 2.2 subgroup in which KRAS mutations are not associated with other recurrent mutations; MAP 3, characterized by *TP53* mutations, subdivided into a MAP 3.1 subgroup in which *TP53* mutations are associated with recurrent *PIK3CA*, *BRAF*, *NRAS*, and *SMAD4* recurrent mutations and a MAP 3.2 subgroup in which *TP53* mutations are not associated with other recurrent mutations; MAP 4, characterized by the absence of *TP53* and *KRAS* mutations, subdivided into a less frequent 4.1 subgroup, characterized by highly recurrent *BRAF* mutations and recurrent *PIK3CA*, *NRAS* and *FBXW7* mutations and a more frequent 4.2 subgroup, characterized by absence of recurrent mutations [25].

BRAF-mutant CRC represent a peculiar subgroup of mCRCs. In the metastatic setting, *^600E^BRAF* mutation occurs in 10% of cases and is associated with a poor prognosis [4]. Among *^V600E^BRAF*-mutated CRCs, two subgroups have been distinguished according either to the activation of KRAS/mTOR/AKT/4EBP1 pathway (BM1 subtype) or to the deregulation in the cell cycle (BM2 subtype) [26]. In addition to *^V600E^BRAF*-mutated CRCs, there is a rarer (occurring in 2% of metastatic CRC patients) subgroup of ^non*V600E*^*BRAF*-mutated CRCs; these ^non*V600E*^*BRAF*-mutated CRCs involve mutation at the level of 19 different codons [27,28]. Patients bearing mutations at the level of codons 594 and 596 seem to form a distinct subgroup with longer overall survival compared with *^V600E^BRAF*-mutated patients [27,29]. A recent study reported the classification of *BRAF*-mutated CRCs into three sub groups: *BRAF* mutations activating RAS-independent as monomers (Class1 V600E); *BRAF* mutations activating RAS-independent signaling as dimers (class 2 codons 597/601); *BRAF* mutations activating RAS-dependent signaling with impaired kinase activity (class 3 codons 594/596) [30]. Class 3 *BRAF*-mutated metastatic CRCs were more frequently left sided and without peritoneal metastases compared to class 1; class 3 tumors have an overall survival comparable to that of *BRAF* wt tumors; while class 1 and 2 tumors have a poorer overall survival than *BRAF* wt tumors [30].

## 3. Comparative Analysis of the Genetic Abnormalities of Primary Metastatic CRCs and of Metastases

Several studies have performed comparative lesion sequencing of paired primary metastatic CRCs and of corresponding metastases.

About 20% of patients with CRC already have metastases at diagnosis [24]. The patterns of metastasis of colon and rectal cancer were recently explored in a very large cohort of patients (49,096, 31,285 with colon cancer and 17,811 with rectal cancer: 30% of colon cancer and 31% of rectal cancer patients had metastases) [31]. Of all patients with metastatic cancer, the most common sites of metastasis were the liver (70% in both colon and rectal cancer) and the thorax (32% in colon cancer and 47% in rectal cancer), followed by the peritoneum for colon cancer (21%) and the bone for rectal cancer (12%); nervous system metastases were more rare, being observed in 5% of colon cancer and 8% in rectal cancer [31]; thoracic metastases were more frequent in lower tumor stages, particularly in rectal cancer, whereas the relative frequency of liver metastases increased with tumor stages; liver metastases were most frequently solitary metastases (in 48% of colon and 45% of rectal cancer); lung metastases were frequently observed in association with liver metastases (73% in colon cancer and 63% in rectal cancer) [31].

Several comparative sequencing studies have shown a high concordance in the genomic profile between primary and metastatic CRCs. Jones and coworkers through a comparative sequencing analysis of a small number of patients observed a high degree of concordance between primary tumors and metastases [32]. Vakiani et al. reported the analysis for *KRAS*, *NRAS*, *BRAF*, *PIK3CA*, and *TP53* genes of 84 CRCs in whom tumor tissue from both primary and metastatic sites was available [33]. The results of this analysis showed that: the frequency of *KRAS*, *NRAS*, and *PIK3CA* mutations was similar in metastatic versus primary tumors; *TP53* mutations were more frequent in metastatic versus primary tumors (53% vs. 30%, respectively), whereas *BRAF* mutations were significantly less frequent (1.9% vs. 7.7%, respectively) [33]. In a subsequent analysis, 69 CRC patients were explored for their mutational profile by NGS in primary and metastatic tumor tissues [33]. The results of this study showed that 79% of the mutations were shared between primary and metastatic tumors. Particularly, a high degree of concordance at the level of early occurring and recurrent mutations was observed [33]. No discordant mutations in *KRAS/NRAS* and *BRAF* were observed; the only private mutations, defined as mutations observed only in the primary or the metastatic tumor, were observed at the level of *APC*, *PIK3CA*, *SMAD4* and *TP53* genes [34]. These findings have supported the view that genetic alterations occurring early during colorectal cancer genesis, such as *APC*, *KRAS*, *NRAS*, and *BRAF* mutations are maintained during the process of tumor evolution up to the final level of tumor metastases [34].

In some contrast with these studies, Vermaat et al., using next generation sequencing, showed a high degree of mutational discordance between primary and metastatic samples, with 52% and 86% of dissimilarities of *KRAS* and *EGFR* mutational status between paired primary and metastatic tumor samples. Modest variability was reported for *HRAS* (34%), *PIK3CA* (19%), *FLT1* (10%), *NRAS* (10%) and *BRAF* (14%) [35].

Lim et al [36] performed an analysis of 34 CRC patients with liver metastases by sequencing (whole exome and RNA sequencing) both primary tumors and metastases and showed in these patients frequent mutations of *APC* (65%), *TP53* (68%), *KRAS* (24%), *TCF7L2* (21%), *PIK3CA* (18%), *NRG1* (18%), *FBXW7* (15%), *SMAD4* (15%), *CARD11* (12%), and *BMI1* (9%) [36]. Based on the absence or presence of mutations in liver metastases, the mutations occurring in these patients were classified into three different classes: class 1, mutations shared between primary tumors and liver metastases (57.6% of all mutations); class 2 mutations present only in primary tumors (20.9% of all mutations); class 3 mutations, detected in only liver metastases (21.5% of all metastases) [36]. Importantly, the frequency of class 1 mutations was highly variable across individual patients (ranging from 25% to 92%), thus suggesting that the presence of a clonal selection during metastasis formation is an event highly variable among patients; a decreased clonality during metastasis formation was usually associated with a high-mutational concordance between primary tumors and metastases, whereas an increased clonality during metastasis formation was usually linked with low mutational concordance between primary tumors and liver metastases [36].

Vignot et al. reported a mutational analysis by targeted NGS on surgical samples from primary and matched metastatic tissues from 13 CRC patients [37]. A global concordance rate for mutations of 78% was observed between primary and metastatic tumors; this concordance raised to 90% for the 12 most recurrent mutations occurring in CRC [37]. On 17 pathways explored, only two pathways were upregulated in metastatic tissues compared to primary tumors [37].

Tan and coworkers reported a detailed analysis of the mutational profile and of CNAs of 18 matched primary and metastatic tumor tissues by high-depth sequencing of over 750 cancer-associated genes and copy number profiling, supporting a high concordance of primary tumor and metastases [38]. Particularly, their results showed a median of 79.3% of somatic gene mutations present both in the primary and metastasis and 81.7% of all alterations present in both primary tumors and metastases [38]. Private alterations, primary-specific or metastasis-specific are observed at lower allelic frequencies [38]. The mutations most frequently occurring only at the level of metastases are represented by *MLL3*, *FAT1*, and *GNAS* gene mutations [38]. Interestingly, distinct mutational signatures are observed in shared variants and private variants [38]. The analysis of copy number alterations similarly showed a conserved pattern between primary tumors and metastases: chromosomal regions of allelic imbalance were similar in the matched primary tumor and metastasis; focal gains and losses of genes commonly amplified or deleted in cancer were similar in the primary tumors and metastases [38]. These findings supported a model of linear evolution in most CRC patients with liver-limited metastatic disease.

Several studies reported a concordant mutation profile for the main CRC driver genes, including *KRAS*, *TP53*, *APC*, *PIK3CA*, *BRAF*, and *NRAS* between primary tumors and metastatic lesions regardless of the temporal relationship between metastases (synchronous or metachronous) [39,40]. Only in a minority of cases (7–15%) metastases differed from paired primary tumors [39,40]. Similarly, Jesinghaus and coworkers have explored the mutational landscape of 24 primary MSS CRCs and of their respective metastases: A high degree of genetic concordance of the mutations affecting the driver genes *APC*, *KRAS*, *FBXW7*, *PIK3CA*, *BRAF*, *SMAD4*, and *ACVR2A* was observed; only 16% of cases displayed the acquisition of new mutations in metastatic lesions involving the *TP53*, *CTNNB1*, *PTEN* and *SYNE1*, all the remaining cases sharing the genetic lesions of the primary tumor with metastases, for all types of metastases, lymph node and distant metastases [41].

Isaque and coworkers have performed a comprehensive whole-genome analysis of differences between metastatic lesions and their corresponding primary tumors in 12 MSS CRC patients [42]. This detailed analysis showed that 65% (range from 36% to 92%) of all mutation events were shared between primary tumors and corresponding metastases, suggesting the existence of a common truncal clone; 15% (range from 1% to 29%) were tumor-specific and 19% (ranging from 3 to 42%) were metastasis-specific; recurrent driver mutations were equally present in primary tumors and their matched metastases, with the exception of only metastatic TP53 mutation, absent in the corresponding primary tumor; a number of metastasis-specific mutations were identified, including non-silent mutations of *FAT1*, *FGF1*, *BRCA2*, *TP53*, and *KDR*, splice site mutations of *JAK2* and 3′-UTR mutations in *KDR*, *PDGFRA*, and *AKT2* genes [42].

Several studies have explored copy number profiles of paired primary and metastatic CRC. Kawamata et al. have analyzed CNAs in paired primary and metastatic tumor samples derived from 16 patients; the CNA profile was explored and was correlated with the timing of primary and metastatic tissue resection and with the exposure to chemotherapy [43]. An average copy number difference of 22% was observed when comparing primary and paired liver metastases; the differences observed between metastases and corresponding primary tumors increased when considering in this analysis post-therapy metastases; some loss of heterozygosity (LOH) events were unique either to primary tumor samples or to metastases: those unique to primary tumors occurred more frequently in those treatment naive, while LOH events unique to metastases occurred most frequently post-therapy [43]. Interestingly, events of amplification of clinically actionable genes *ERBB2*, *FGFR1*, *PIK3CA*, or *CDK8* were observed in some patients at the level of metastases but not in the corresponding primary CRCs [43].

Smeets and coworkers investigated the pattern of CNAs in 409 metastatic CRC patients undergoing treatment with chemotherapy alone or chemotherapy plus bevacizumab in the context of the phase II MoMa study [44]. mCRCs were clustered into three different subgroups according to increasing degrees of chromosomal instability: tumors belonging to the intermediate-to-high instability subgroups have improved outcome following treatment with chemotherapy plus bevacizumab versus chemotherapy alone; low instability tumors, including POLE-mutated and MSI tumors, derive no further benefit from bevacizumab [44].

The targeted therapy of metastatic CRC patients implies the exploration of the targeted biomarker and its presence in both primary and metastatic tumors. The introduction of EGFR inhibitors for treatment of metastatic CRC patients allowed the unique opportunity to obtain, through the analysis of numerous clinical studies, data on the concordance of the mutational status for *KRAS*, *NRAS*, *BRAF* and *PIK3CA* between primary tumors and metastases in more than 3500 patients [45]. This metanalysis involving 61 clinical studies and data on 3565 metastatic CRCs showed: (i) a very high median biomarker concordance for *KRAS* (93%), *NRAS* (100%), *BRAF* (99.4%), *PIK3CA* (93%); (ii) a pooled discordance of 8% for *KRAS*, 8% for *BRAF*, and 7% for *PIK3CA* [35]. These observations further support the maintenance of the main driver mutations in CRCs undergoing metastatic spreading [45].

## 4. Tumor Heterogeneity and Metastatic Evolution

Study of intratumor heterogeneity (ITH) is fundamental from both a biological and clinical perspective, to understand the genomic changes driving the evolution of the malignant process up to metastasis generation. Several studies have shown that CRCs display a consistent degree of spatial intratumor heterogeneity; particularly, three types of spatial heterogeneity of CRCs have been described: (i) ITH related to the existence of genetic differences at the level of tumor cells within the primary tumor; (ii) ITH related to differences at the level of various metastatic lesions within a single patient; (iii) ITH related to the existence of genetic differences within the cells of a single metastatic lesion (intrametastatic heterogeneity) [46].

An initial study by Baisse and coworkers provided evidence through multiregional sequencing analysis of 15–20 areas within a tumor, that 67% of advanced CRCs displayed significant ITH at the level of gene alterations and CNAs [47]. Jeantet and coworkers performed the analysis of the distribution of *RAS* mutations in different areas of primary tumor, metastatic lymph nodes and distant metastases: primary tumors displayed an intra-tumoral heterogeneity for *RAS* mutations in 33% of cases; the comparative analysis of primary tumors and metastatic tumors showed an inter-tumoral heterogeneity in 36% of cases; multiple *RAS* mutated subclones were observed in 28% of cases in the same tumor [48].

Kim and coworkers have performed a multiregion analysis of the mutational spectrum and CNAs at the level of both primary and metastatic colorectal cancer lesions from five CRC patients [49]. This study showed a substantial level of ITH in both primary and matched liver metastases, with 46% to 80% subclonal mutation fractions. The spatial localization of the mutations allowed their classification into three types: the universal mutations are those observed in all the regional biopsies, are enriched in genes such as *APC*, *KRAS*, and *TP53* and represent events occurring early during tumor evolution; metastasis-clonal mutations are those that are regionally clonal only in the metastatic regions and may represent genetic events involved in the development of distant metastases; primary-private mutations are those present in primary but absent at the level of metastases; metastasis-private mutations are those present in primary but absent in only a part of metastatic lesions and may represent events that are acquired during the expansion of metastatic clones [49]. It was estimated that 20–54% of mutations in a given sample were universal, whereas from 46% to 80% of mutations were subclonal; among the subclonal lesions, 1–15% were metastasis-clonal, 2–41% metastasis-private, and 14–56% primary-private [49]. Most CNAs containing genes involved in CRC development, such as *APC*, *PTEN* and *SMAD4* were observed in both primary and metastatic lesions, thus representing early or universal genomic events [49]. In contrast, copy number changes such as chromosomal gains of *c-MYC* and chromotripsis can be region-specific and may represent the source of genetic intra-tumor heterogeneity. Finally, the inferred evolution pattern of cancer progression was as a branched evolution, rather than as linear evolution [49].

Sveen et al [50] have reported high-resolution DNA copy number analysis of metastatic lesions from 45 CRC patients; this analysis showed a pronounced variation in the level of intra-patient inter-metastatic heterogeneity [50]. Interestingly, the level of intra-patient inter-metastatic heterogeneity resulted to be a strong prognostic determinant, stronger than commonly adopted clinico-pathological prognostic markers: patients with a high-level of heterogeneity had a three-year overall survival of 18%, compared to 66% for patients with a low-level of tumor heterogeneity [50].

Uchi and coworkers have investigated intratumor heterogeneity in CRC by analyzing samples from distinct areas of 9 different primary tumors [51]. Multiregional exome sequencing provided evidence about the existence of extensive intratumor heterogeneity and branched evolution. Particularly, the analysis of the various mutations showed that they can be classified as founder, shared and unique mutations: parental clones acquire mutations in driver genes, such as *APC*, *KRAS* and *FBWX7* as founder mutations during tumor development, whereas subclones acquire mutations in *PIK3CA* mutations as progressor mutations [41]. The age of patients correlated with the number of founder mutations. Similar to gene mutations, some copy number alterations occurred as founder events (such as amplifications of 7p, 13q, 10q, 20p, and 20q), while other CNAs, such as several focal deletions, predominantly occur as progressor CNAs [51]. The analysis of epigenetic intratumoral heterogeneity showed that CIMP-H occurs early in tumor evolution [51]. Similar to the other genetic alterations, some epigenomic modifications occurred as founder events, such as hypermethylation of *SFRPs*, *GATA4* and *GATA5* genes, whereas other epigenomic modifications occurred as progressor events [51]. An integrated view of the various parameters of intratumor genetic/epigenetic heterogeneity allowed the reconstruction of each CRC’s life history. A typical example is given by one of these nine patients: in this patient, the initial founder mutations, *APC*, *KRAS* and *FBWX7* mutations, were observed at the level of the parental clone; this initial parental clone subdivided into two subclones, one characterized by the acquisition of a focal *MYC* amplification and the other one by several shared CNAs, such as 20p amplification and 1p deletion. At the subsequent steps of tumor evolution, the two subclones branched into minor subclones, a process accompanied by accumulation of progressor mutations and methylation alterations. These events caused the development of a consistent degree of intratumor heterogeneity, extended also at the level of transcriptome heterogeneity [51]. Interestingly, these authors have performed a comparative analysis of ITH in early and advanced CRCs, providing evidence that early tumors acquire more subclonal driver mutations compared to advanced tumors: in early CRCs 50% of driver mutations were branch mutations, while only 22% mutations were branch mutations in advanced colorectal cancers [52].

Some studies have explored ITH of CRCs using deep sequencing techniques. Thus, Wei et al. performed a high depth multiregional wide exome sequencing in 28 tissues from four CRC patients with matched primary and metastatic tumors. This study provided several interesting findings to better understand the process of CRC metastasization: metastatic tumors exhibited less intratumor heterogeneity than primary tumors; primary and metastatic tumors differ significantly based on the analysis of allelic frequency of the various mutations; all metastatic tumors inherited multiple genetically distinct subclones from primary tumors, thus suggesting a possible polyclonal seeding mechanism for metastasis [53]. In one of these patients, both lymph nodes and lung metastases were analyzed, showing a completely different genetic landscape in these two different metastatic sites; according to this finding, it was suggested that parallel metastatic dissemination to distant organs is independent of lymph nodes [53]. Suzuki et al. have shown a variable level of ITH using deep-targeted NGS followed by ultra-deep amplicon sequencing through the analysis of 4 different CRC patients investigated at the level of various tumor regions; different tumor regions shared mutations in driver genes, such as *APC*, *KRAS* and *TP53*. However, in addition, many mutations were observed only at subclonal levels and in many instances their detection was only revealed by an ultra-high-depth sequencing approach [54].

Very interestingly, Oh and coworkers performed a study of intratumor heterogeneity on a large set of patients across 8 different tumor types by targeted deep sequencing; using this technique, a ITH index was determined showing that CRCs are among the tumors with the highest ITH index [55]. In this study, CRC patients of all tumor stages were included showing that ITH index was already high in 40% of stage I patients and moderately increased with tumor stage progression, with a high ITH index in 55% of stage IV CRCs [55]. The presence of high ITH index was clearly associated with a decreased progression-free survival (PFS) in stage I-III patients, but not in stage IV patients [55].

It is important to note that intratumor heterogeneity is not dictated only by genetic mechanisms, but also by phenotypic heterogeneity/plasticity apparently unrelated to genetic determinants. A notable example is provided by a study by Kreso et al., based on the analysis of serially expanded CRC clones from patient samples, remaining genetically stable during serial transplantation; in spite this stability, reproducible differences in the functional fates and response to chemotherapy of individual CRC cells, suggesting that in vivo dynamic changes of CRCs are not dictated by genomic changes [56]. These observations support the view that, in addition to the well-known mechanisms of tumor heterogeneity driven by genetic diversity, other diversity-generating processes exist within a genetic clone, seemingly related to epigenetic diversity, variability of tumor microenvironment and multiple external factors affecting gene expression [56].

## 5. Liver Metastases

The liver is the most frequent metastatic site for CRC, with 60% of CRC patients developing colorectal liver metastases (CLMs). CLMs can be surgically removed or therapeutically ablated and these procedures may significantly improve the survival of these patients. Recent studies have explored a possible link between genomic features and outcomes of metastatic CRC undergoing CLM resection. Initial studies have suggested that mutations in *KRAS* and *BRAF* are associated with a poor outcome after CLM resection, whereas mutations in *NRAS*, *TP53*, *PIK3CA*, and *SMAD4* were shown to be potential prognostic factors after CLM resection [57].

More recent studies have shown that the analysis of co-mutation status is more predictive of outcome after CLM removal [57]. Thus, it was shown that *RAS/TP53* double-mutant metastatic CRC with predominant location in right colon of primary tumors, corresponding to 31% of patients, displayed a shorter five-year overall survival (12%), compared with 55% overall survival of *TP53* wild-type [57].

The presence of *V600E BRAF* mutations observed in 5.1% of metastatic CRC patients, but not non-*V600E BRAF* mutations was associated with worse prognosis (reduced survival and frequent and rapid recurrence) after resection of CLMs [58]. Interestingly, *V600E BRAF* mutations had a stronger association with overall and disease-free survival than *KRAS* mutations [58].

Datta and coworkers explored a large group of 935 patients with metastatic CRC and showed that co-alteration of oncogenic *TP53* with either *KRAS*, *NRAS*, or *BRAF* mutations was associated with significantly worse survival compared to alterations in either gene group alone [59]. Interestingly, *RAS/BRAF-TP53* co-mutated CRCs were associated with worse survival in patients with liver and lung, but not with peritoneal surface metastases Moreover, co-altered *BRAF/RAS-TP53* were significantly associated with the development of extra-hepatic metastatic sites [59]. Similar conclusions were reached by Kawaguchi et al. who analyzed the possible relationship between somatic gene mutation profile and outcome in 507 metastatic CRC patients who underwent CLM resection: *BRAF*, *RAS*, *TP53*, and *SMAD4* mutations were significantly associated with overall survival, coexisting mutations in *RAS*, *TP53*, and *SMAD4* were associated with negative outcome (reduced OS and RFS) than coexisting mutations in any two of these genes and mutations in one or more of these genes [60].

Smith et al. recently reported the results of a retrospective study on 370 metastatic CRC patients who underwent either colorectal liver hepatectomy followed by hepatic arterial infusion (HAI) chemotherapy or HAI and systematic therapy (patients with unresectable metastases); 34.8% of these patients have extrahepatic disease and 65.2% have liver-restricted disease [60]. Concurrently mutated *RAS/BRAF* and *SMAD4* were associated with negative survival in resectable patients, while concurrent *RAS/BRAF* and *TP53* mutations were associated with worse survival in unresectable patients [61].

Leung et al. have developed a highly multiplexed single-cell DNA sequencing to trace the metastatic lineage of two CRC patients with matched liver metastases [62]. In the first patient, a monoclonal seeding was observed, in which a single clone of tumor cells acquired a large number of mutations before developing the capacity to migrate to the liver and to develop an advanced, metastatic tumor; in the second patient, a polyclonal mechanism of seeding was observed, in which two clones that have diverged from the primary tumor metastasize to the liver [62]. Interestingly, the single-cell sequencing approach allowed to show the existence in one of the two patients of a rare subpopulation of diploid cells that carried a heterozygous mutation in *APC* gene, but not associated with other somatic mutations; these cells were diploid and seemingly represent the initial tumorigenic cells and remained present in the advanced tumor representing 2.6% of tumor cells [62]. A second unexpected finding was observed in the second patient and consisted in the detection of a small independent subpopulation of diploid tumor cells that harbored a completely different set of mutations than the main tumor lineage [62].

## 6. Lymphatic Metastases

Other studies have explored the process of CRC metastasization, focusing on the mechanisms of spreading of cancer cells from the primary tumors to regional lymph nodes. Lymph node metastasis associates with negative outcomes in CRCs and the presence of tumor cells in regional lymph nodes defines stage III disease and the need for adjuvant chemotherapy and lowers the 5-year survival compared to stage II disease without lymphatic lymph nodes metastasis [63].

Naxerova et al. have explored the evolutionary relationship between primary tumor, lymph node and distant metastases in CRC: through the study of 213 biopsy samples from 17 patients, these authors have used somatic variants in hypermutable DNA regions to reconstruct phylogenetic trees of tumor metastatic evolution [64]. This analysis provided evidence about the existence of two different pathways of lymph node and distant metastases generation in CRC patients. In fact, the genetic distances between lymph node metastases, distant metastases and corresponding primary tumors were measured showing that for the majority (73%) of lymph node metastases the distance with respect to the primary tumor was shorter than the distance with respect to distant metastases; distant metastases (69% of cases) had shorter distance to the primary tumor than to lymph nodes metastases [64]. In line with these observations, reconstruction of phylogenetic evolutionary tumor trees allowed to establish that in 35% of cases lymphatic and distant metastases have a common origin from the same subclone of the primary tumor (either they originate both from the primary tumor or, alternatively, distant metastases originate from lymph node metastases). In contrast, in 65% of cases, there is evidence of a distinct origin of lymphatic and distant metastases, as supported by the evidence of genetically different alterations, thus indicating that in these patients primary tumors harboring multiple subclones at different stages of evolution have seeded genetically distinct metastases [64].

Ulintz and coworkers have explored the clonal origin of lymph node metastasis in CRC. Thus, they have investigated multiple tumor regions and cancer-containing lymph nodes from 7 CRC patients, providing evidence that: (i) for each patient, the primary tumor regions and matched lymph node metastases were polyclonal and the clonal populations differed from one node to another; (ii) in a part of CRC patients, the cancer cells present in a given lymph node originated from multiple distinct regions of a primary tumor, while in other cases these metastatic cells originate from a single geographic region of the primary tumor; (iii) lymph node metastases contain subclones originated early or late during tumor development [65]. According to these findings, a model of lymph node metastatic spreading in CRCs involving multiple waves of seeding from the primary tumor over time was proposed [65].

Hu et al. have recently characterized the evolutionary dynamics of metastatic seeding by analyzing exome sequencing profiles from 118 biopsies derived from 23 patients with CRC with metastases to liver or brain [66]. Particularly, these authors performed multi-region sequencing on the primary tumor and paired metastasis to build phylogenetic trees. The results of this study indicate that the genomic divergence between the primary tumor and paired metastases is low: mutations in *KRAS*, *TP53*, *SMAD4*, *TCF7L2*, *FN1*, *ERLF3*, and *ATM* were highly concordant between primary tumors and metastases and 70% of highly frequent gene mutations were shared by both lesions, a finding similarly observed in liver and brain metastases; among the genes that tended to be private to the primary tumors or to metastases the most frequent were *SYNE1* and *APOB*. Somatic copy number alterations were generally concordant. Some putative oncogenes, such as *PIK3CA*, *GNAS*, *SRC*, *FXR1*, *MUCA*, *GPC6*, and *MECOM* were more recurrently amplified in metastases than in primary tumors [56]. Interestingly, the analysis of genetic data relative to large sets of CRC patients allowed to define the existence of metastasis-associated early driver gene modules present in early tumors and characterized by modules of tumor cells exhibiting CRC drivers (combinations of *APC*, *KRAS*, *TP53*, or *SMAD4*) associated with potential metastasis-associated genes, such as *TCF7L2*, *AMER1*, or *PTPRT* [56]. Interestingly, *PTPRT* mutations in combination with canonical CRC drivers are almost exclusively found in metastatic CRC patients [66]. The simulation of spatial tumor growth under selective or neutral growth evolutionary modes, coupled with the evaluation of the patterns of subclonal divergence at the level of different tumor regions allowed to establish whether a given tumor is driven by positive selective selection (either strong or weak) or by neutral evolution. The development of a spatial computational model of tumor progression and statistical inference framework to time dissemination in a patient-specific fashion, allowed to suggest that the capacity to seed metastasis is a property inherent to cancer cells originated early during tumor development (81% of cases), when the tumor bulk is clinically undetectable [66]. The analysis of a large set of public databases provided evidence that the large majority (90%) of metastatic primary CRCs displayed subclonal selection, thus suggesting that the metastatic clone possesses a consistent selective growth advantage. However, only a lower proportion (33%) of stage I-III CRCs displayed patterns of tumor evolution compatible with subclonal selection. Importantly, this observation suggests that type of tumor evolution may be dependent on disease stage or disease aggressiveness [66]. As mentioned above, driver mutations were usually not enriched in metastases; however, the stratification of CRC patients according to the profile of tumor evolution (early dissemination vs. late dissemination) showed a higher frequency of private driver mutations in metastases evolving under selection conditions compared to those evolving neutrally, thus suggesting that in these patients additional subclonal driver mutations may occur during the development of some metastases [66].

The same authors very recently reported the analysis of whole-exome sequencing data from 457 paired primary tumors and metastases derived from 136 patients with colorectal, breast and lung cancer: this study involved the analysis of 39 metastatic CRC patients, including both untreated and treated metastases [67]. The results of this study provided several interesting findings: (i) the mutational burden (single nucleotide variation and CNAs) was highly concordant between primary and metastatic tumors; (ii) metastases displayed a slight increase in the number of clonal single nucleotide mutations and fewer subclonal nucleotide variants, supporting the existence of an evolutionary bottleneck during metastasis; (iii) a high percentage (84%) of clonal drivers in each primary CRC tumor and metastasis was shared, while the fraction of subclonal drivers was 20%; (iv) among the three cancers investigated, CRC had the highest prevalence of primary tumor-private subclonal drivers; (v) driver mutations present in metastases are enriched in the trunk of the phylogenetic mutational tree; (vi) treatment induced a dramatic increase of the frequency of private clonal drivers across all the three cancers, including CRC (78% of metastasis-private clonal driver mutations), thus suggesting that therapy selects a minor micro metastatic subclone; (vii) a small number of driver genes that were more frequently amplified or deleted in metastases compared to primary tumors (such as amplification of *RAC1* or deletion of *FAT1* and *ALB* genes); (viii) polyclonal seeding was common in untreated lymph node metastases and distant metastases, but was less frequent in treated distant metastases [67]. The low number of metastasis-private clonal mutations is consistent with early metastatic seeding [67].

## 7. Effect of Therapy on Mutational Landscape of Metastatic CRC

The targeted therapy of metastatic CRC patients implies the exploration of the targeted biomarker and its presence in both the primary and the metastatic tumors. The introduction of EGFR inhibitors for treatment of metastatic CRC patients allowed the unique opportunity to obtain, through the analysis of numerous clinical studies, data on the concordance of the mutational status for *KRAS*, *NRAS*, *BRAF* and *PIK3CA* between primary tumors and metastases in more than 3500 patients [45]. This meta-analysis involving 61 clinical studies and data on 3565 metastatic CRCs, showed: (i) a median biomarker concordance for *KRAS* (93.7%), *NRAS* (100%), *BRAF* (99.4%), and *PIK3CA* (93%); (ii) a pooled discordance of 8% for *KRAS*, 8% for *BRAF*, and 7% for *PIK3CA* [45]. These observations further support the maintenance of the main driver gene alterations in CRCs undergoing metastatic spreading [45]. The detection of *KRAS* mutations in metastatic CRC is important because implies a negative prognosis and a poor response to standard chemotherapy [68].

An important example of the therapy-driven effects on the genomic alterations of metastatic CRC derives from the analysis of patients developing resistance to therapies based on EGFR inhibitors. EGFR inhibitors are effective in a subset of *KRAS* wild-type metastatic CRCs; however, after an initial response, the development of secondary resistance mechanisms cause disease relapse, thus limiting the clinical benefit of this treatment: The analyses of metastases of patients who developed resistance to EGFR inhibitors showed more rarely the emergence of *KRAS* amplification and more frequently the acquisition of secondary *KRAS* mutations; in these patients, *KRAS* mutant alleles were detectable in the blood circulating tumor DNA 10 months before the radiographic documentation of disease progression [69]. These observations suggest that EGFR-targeted therapy exerts a selective effect on CRCs either inducing the expansion of pre-existing *KRAS*-mutant subclones or favoring the development of new *KRAS* alterations [69]. Another mechanism of secondary resistance to EGFR blockade is represented by novel alterations of ectodomain of EGFR [70]. The study of individual patients has shown that different metastatic biopsies from the same patient with CRC display genetically distinct mechanisms of resistance to EGFR blockade: thus, in some patients, it was documented that distinct resistance mechanisms emerge in different metastases in the same patient and can drive lesion-specific responses to different targeted therapies [70].

Genetic mechanisms of primary resistance to EGFR inhibitors among *KRAS* wild-type CRC patients are represented by *NRAS* mutations, *^V600E^BRAF* mutations, *MET* amplification, *ERBB2* amplification, *PIK3CA* mutations at the level of exon 20, mutations in *FGFR1, PDGFRA*, and *MAP2K1*, and homozygous deletions of *PTEN* [71].

Using xenografts derived from hepatic metastases of CRC patients, amplification of *ERBB2* was identified as a potential therapeutic target in cetuximab-resistant CRCs [72]. These preclinical observations supported a clinical study (HERACLES) evaluating trastuzumab and lapatinib in metastatic CRC patients with amplified *ERBB2* refractory to standard cares: in 33 patients, 24.2% objective responses were observed with durable clinical benefit lasting >24 months in responding patients [72]. Although ERBB2 blockade was effective, most of responding patients relapse [73]. A recent study explored the mechanisms of tumor evolution responsible for relapse to HER2 blockade. In fact, the analysis of circulating tumor DNA allowed to define organ and metastases-private evolutionary patterns and high-levels in intra-patient molecular heterogeneity, defining lesion-specific evolutionary trees and potential pharmacologic vulnerabilities [74].

## 8. Models of CRC Progression and Evolution

The study of tumor heterogeneity is a fundamental tool to analyze and to define the molecular and cellular mechanisms responsible for the development of CRC and have provided a consistent contribution to the development of current theories to explain CRC development.

Two different models have been proposed in the time to explain the origin and development of CRC metastasis: one suggesting a common origin for both the primary tumor and metastases and the other hypothesizing a completely independent genesis of metastases and of the primary tumor. The sequencing data of matched primary tumors and metastases have strongly supported the existence of a common ancestor of both the primary tumor and of the corresponding metastases.

The development of CRC from a common ancestor implies two different models to explain metastasis evolution: the parallel progression model suggests that the dissemination of metastasizing tumor cells occurs during early stages of primary tumor and the primary tumor and metastases evolve separately thereafter. The linear progression model implies the occurrence of metastases as a sequential event occurring during primary tumor development.

### 8.1. Somatic Mutations in Normal Colonic Epithelium

Colon epithelium is organized in crypts, composed by about 2000 cells, representing the tissutal units. The main function of crypts consists in providing an efficient system of renewing of the short-lived colonic epithelium, through the differentiation of intestinal stem cells, located at the base of the crypts; these stem cells stochastically replace one another through a biologic process of neutral drift, thus ensuring that all stem cells and differentiated cells present in a crypt derive from a single ancestral stem cell. As a consequence of this hierarchical organization of the intestinal crypt, somatic mutations in these ancestor stem cells are present in all the stem cells composing the crypt; these stem cells are considered the cells of origin of CRCs [75]. A recent study explored somatic mutational landscape in normal colorectal epithelium through whole-genome sequencing of normal colorectal crypts from 42 individuals [76]. Signatures of multiple mutational processes were detected, with some signatures being ubiquitous, while other ones observed in some individuals, in some crypts. Driver mutations were observed in about 1% of normal colorectal crypts in middle-aged individuals [76]. Among the driver mutations detected in normal crypts there are *AXIN2*, *STAG2*, *PIK3CA*, *ERBB2*, *ERBB3*, *FBXW7* mutations [66]. A different pattern of mutations was observed in normal crypts compared to those observed in CRCs: *ERBB2* and *ERBB3* mutations are common in normal colon but rare in CRCs (1%), whereas mutations in driver genes mutations in *APC*, *KRAS* and *TP53* are common in CRCs, but are rare among normal crypts (one in 14) [66]. These observations strongly suggest a major oncogenic potential to *APC*, *KRAS*, and *TP53* mutations promoting the conversion to colorectal adenoma (CRA) and CRC, whereas mutations in *ERBB2* and *ERBB3* confer higher like hoods of crypt colonization by stem cells [76]. No significant difference was observed in the frequency of driver mutations between individuals who had CRC and those who did not [76]. According to these findings, it was concluded that CRAs and CRCs are rare outcomes of a pervasive process of neoplastic change occurring at the level of morphologically normal colorectal epithelium [76].

The investigation of individuals with inflammatory bowel disease provided evidence that the repeated inflammatory cycles affecting the colonic epithelium induce a 2.4-fold increase of the average rate of colonic crypts affected; the mutations observed in IBD non-neoplastic epithelium mostly involve *ARID1A*, *FBXW7*, *PIGR*, and *ZC3H12A* genes in the IL17 and Toll-like receptor pathways [77]. Mutations in *KRAS*, *APC* and *TP53* are rare in non-dysplastic tissues from IBD patients. At variance with the normal colon, where clonal expansions are limited to the crypts, in IBD epithelium, frequent widespread millimeter-scale clonal expansions were observed [77]. The differences in driver landscape of IBD colon, suggest that there are different selection mechanisms in the colitis-affected colon and that somatic mutations potentially play a causal role in IBD pathogenesis [77].

Nicholson and colleagues have analyzed stem cell dynamics in normal human colon to define the efficiency of clone fixation within the epithelium and the rate of subsequent lateral expansion [78]. The process of mutant clone fixation within colonic crypts takes years, due to the time required for the mutated intestinal stem cells to replace neighbors cells to populate the entire crypt; crypt fission allows the lateral expansion of mutant clones: this process is rare for neutral mutations (0.7% per year); biases in both fixation and expansion of stem cells increases age-related pro-oncogenic burden; pro-oncogenic mutations modify the stem cell turnover and accelerate fixation and clonal expansion by crypt fission to generate high mutant allelic frequencies with age [78].

### 8.2. Mutational Landscape in the Progression from Colorectal Adenomas to Colorectal Cancers

Several studies have compared the spectrum of genetic alterations in CRAs and in CRCs.

In an initial study, Jones et al. have performed an analysis of the mutations observed in benign, invasive and metastatic colorectal tumors and reached the conclusion that more selective mutational events are required for the transition of a benign adenoma into a CRC than those required for the acquisition of metastasizing properties by a CRC [32]. The results of this study supported a classical model of colorectal tumorigenesis, characterized by the progressive acquisition of mutational events through various clinical stages of tumor progression: the tumor process is initiated by the acquisition of a mutation into a gene of the Wnt pathway (mostly *APC* mutations) with consequent formation of a small adenoma; mutations constitutively activating *KRAS/BRAF* pathway are required for the proliferation of the small adenoma and for its transformation into a large adenoma; subsequent acquisition of mutations at the level of genes controlling the PIK3CA, TGF-β and TP53 pathways is required for the transformation of a benign adenoma into a CRC; only few metastasis-specific mutations are acquired during the transition of a CRC from an invasive condition to a metastatic status [32].

*APC* loss of function is a key event in the colon carcinogenesis and represents the first event in the tumor initiation. This conclusion was directly supported through sequencing studies on colon adenomas. Nikolaev and coworkers have performed an exome sequencing analysis of 24 human colon polyps, derived from 22 individuals with no family history of predisposition to cancer. The mutational profiles observed at the level of the cancer-driver genes *APC*, *CTNNB1* and *BRAF* genes allowed to subdivide polyps into three different groups: the group 1 with *APC* mutations, included the majority of polyps, mostly corresponding to colon adenomas: All the observed mutations introduced premature stop codon and none of these polyps retained a normal *APC* allele, due to the presence of two *APC* mutations or a single *APC* mutation associated with loss of heterozygosity; the group 2 with *CTNNB1* mutations included only a few minority of polyps: the *CTNNB1* mutation was homozygous, due to concomitant LOH; the group 3 with *BRAF* mutations included polyps with serrated histology: *BRAF* mutations were heterozygous [79]. Adenomas with *CTNNB1* or *BRAF* mutations did not display mutations in other cancer-driver genes, whereas adenomas with *APC* mutations showed additional cancer-driver mutations (at the level of *KRAS*, *NRAS*, *GNAS*, *AKT1*, *SOX9* and *TP53* genes), whose number correlated with the degree of dysplasia and invasiveness [79]. In addition to cancer-driver gene mutations, many passenger mutations were observed in colon adenomas [69]. According to the rate of single nucleotide substitutions, it was suggested the existence of a mutator phenotype in colon adenomas [79].

Lin and coworkers have reported the results of a whole-exome sequencing and targeted sequencing study on 149 colon adenocarcinoma samples, corresponding to 134 conventional adenomas (CADs) (104 non-advanced and 30 advanced) and 14 serrated adenomas (SSAs). No significant differences in the mutation rates were found between CNADs and SSAs (1.5 and 1.7 mutations/Mb, respectively) [80]. As it is expected, the gene most frequently mutated in CNADs was *APC*, while *BRAF* was the gene most recurrently mutated in SSAs [70]. In addition to *APC*, four genes were frequently mutated in CADs: *CTNNB1* (catenin beta 1), *KRTAP4-5* (keratin-associated protein 4-5), *GOLGA8B* (golgin A8 family member B) and TMPRSS13 (transmembrane protease, serine 13) [80]. The biological role of *GOLGA8B*, *TMPRSS13* and *KRTAP4-5* in the development of colon adenomas and in their progression to CRC remains largely unknown. The comparison of the mutational profile observed in non-advanced CADs, advanced CADs and CRCs showed that: *PIK3CA* and *SMAD* mutations are absent in CADs; *APC* mutations are increasing from non-advanced to advanced CADs; *KRAS* and *TP53* mutations are progressively increasing in the progression from non-advanced to advanced CADs and then to CRCs [80]. The identification of some CRC-specific mutated genes, absent in CADs, provides a tool for distinguishing between adenomas and CRCs and supports the view that some mutational events are essential for the transition from benign adenomas to CRCs [80].

Lee and coworkers reported the mutational profiling by whole-exome sequencing of 12 high-grade colon adenomas (HGCAs, 11 non-hypermutated and 1 hypermutated). This analysis showed that total numbers and spectrum of somatic mutations detected in HGCAs were not consistently different from those observed in CRCs [81]. The most recurrent gene alterations observed in these tumors consisted in mutations of *APC*, *KRAS*, *SMAD4*, *ERBB4*, *AMER1*, and *TP53* genes, copy number loss of *SMAD4*, and copy number gain of *GNAS* and *ARID2* genes [81]. The peculiar finding of this study was related to the observation that mono-allelic inactivation of *SMAD4* may occur in HGCAs.

Druliner and coworkers have recently reported the analysis of cancer-adjacent polyps (CAPs) and cancer-free polyps (CFPs): CAP cases included matched, distant normal colon epithelium, the polyp (residual polyp of origin) and the corresponding cancer that arose from the polyp, whereas CFP cases include matched, distant normal colonic epithelium and colon adenoma (polyp) [82]. The mutational spectrum of CAPs and CFPs was explored by wide exome sequencing; the majority of the top 10 genes involved in CRC tumorigenesis had a mutational frequency higher in CAPs than in CFPs: *TP53*, *FBXW7*, *PIK3CA*, *KIAA1804*, *SMAD2*, and *SMAD4* were almost exclusively mutated in CAPs [82]. Thus, the CAPs displayed an increased number of genetic variants as compared to the CFPs and the genes preferentially or exclusively mutated in the CAPs were enriched for cancer pathways [82]. Some genes, *GREM1*, *IGF2*, *CTGF*, and *PLAU* displayed significant changes between CFPs and CAPs [82].

In a recent study, Cross and coworkers have mapped the evolutionary landscape of CRAs and CRCs through the study of multi-targeted whole genome sequencing on 2–16 regions from 9 CRAs and 15 CRCs [83]. The mutational frequency (single nucleotide alterations) was similar in CRAs and CRCs; the burden of driver mutations was similar in CRAs and CRCs. Individual driver gene mutations were detected at similar frequencies across CRAs and CRCs, with the exception of TP53, which was more commonly mutated in CRCs than in CRAs [83]. Intra-tumor heterogeneity and phylogenetic analyses suggest that CRCs occupy sharper fitness peaks that CRAs: 56% of CRA single nucleotide alterations (SNAs) were subclonal, while only 45% of CRC SNAs were subclonal. The phylogenetic trees of CRAs have shorter trunks and longer branches/leaves than those of CRCs; CRAs were more heterogeneous than CRCs, suggesting that the former occupy a broader fitness peak than the latter ones [83]. The analysis of non-synonymous mutations to synonymous mutations on the branches/leaves of CRCs relative to their trunks, but not of CRAs, possibly suggesting a possible positive subclonal selection in CRAs; these findings suggest that subclonal selection is absent/weak at the level of established CRC [83]. The driver gene alterations can be subdivided into tier 1 mutations (mutations or gene alterations playing a defined role in CRC pathogenesis) and tier 2 mutations (gene alterations of uncertain pathogenic role or pan-cancer genes): tier 1 driver mutations were very frequently clonal in both CRAs (80%) and CRCs (89%); tier 2 driver mutations were less frequently clonal in CRAs (47%), compared to CRC (80%) [83]. The analysis of copy number alterations showed some remarkable differences between CRAs and CRCs: Adenomas had fewer CNAs than CRCs and the overall average proportion of the genome disrupted by CNAs was lower in adenomas (40%) than in CRCs (72%) [83]. Driver CNAs in CRC involve losses of chromosomes 5q (*APC*), 17p (*TP53*) and 18q (*SMAD4*): 17p loss occurred more frequently in CRCs than in CRAs, whereas loss of 5q and 18q occurred at similar frequencies in CRAs and CRCs [83]. 78% of CN gains were subclonal in CRAs compared to 48% in CRCs; 57% and 27% of CN losses were subclonal in CRAs and CRCs, respectively [83]. The evolution of CRC involves either a punctuated or a more gradual CNA acquisition [83]. Finally, the analysis of few MSI^+^ CRCs indicate that these tumors evolve in a similar way to MSS CRCs, with a higher mutational burden and with a more limited evidence of subclonal selection [83]. The ensemble of these observations suggests that CRAs can harbor mutations in any CRC driver gene and driver acquisition does not necessarily involves selective sweeps, inducing stepwise evolution of the tumors, as supported by the finding that subclones with additional driver mutations do not replace subclones lacking these driver mutations, but co-exist in different areas of the tumors [84].

### 8.3. The Classical Linear Progression Model and the Big Bang Model

The classical linear progression model implies that a CRA is initiated by two genetic alterations at the level of the *APC* gene and progresses to invasive CRC through a progressive, stepwise acquisition of additional genetic alterations involving driver gene mutations such as *KRAS* and *TP53* and deletion of chromosome 18q4 [74]. The evolutionary dynamics of this process is governed by a series of progressive selective sweeps to fixation, each involving the progressive development of subclones exhibiting increasing fitness, due to the acquisition of new driver mutations [32].

Using early index-lesion sequencing and a mathematical model helping to translate the mutational events into distance of time, it was estimated a shorter time required for the development of metastases from advanced CRC (1.8 yeas) than for the development of an advanced CRC from a colon adenoma (17 years) [32].

In 2015, Sottoriva and coworkers proposed the “Big Bang” model of human colorectal tumor evolution, based on the assumption that these tumors are genetically heterogeneous from their initiation and subsequent genetic alterations are changes of their original ancestral cancer-driving alterations [85]. Several observations support the Big Band model: (i) Intratumor heterogeneity is a “constitutive” property of CRCs arising from their initiation and increasing with their progressive growth, not significantly influenced by events of clonal selection; this spontaneous propensity to intra-tumor heterogeneity predisposes the CRCs to a branched phylogeny pattern of growth. (ii) Marked clonal expansions or selective sweeps are rare events at the level of CRCs at an advanced stage of tumor development. (iii) Both universal and private genetic alterations originate early during tumor development a become widespread during tumor progression, thus becoming the dominating elements in the genetic structure of developed CRCs. (iv) Aggressive subclones are present in the primary tumor and remain rare; however, these subclones have a relative fitness advantage that contributes to fuel resistance to drug treatments and may become dominant under these circumstances [85].

Several observations directly support this theory. In fact, Kang et al [86] have explored the mutational heterogeneity of colorectal adenomas and reached the conclusion that these tumors display the presence of private mutations in different parts of the same tumor. This consistent intratumor heterogeneity originates from the first tumor divisions [86]. Sievers have investigated the mutational landscape of small colorectal polyps and showed that these tumors carried 0–3 driver pathogenic mutations, the most frequent being *APC*, *KRAS*, *TP53*, *BRAF*, *FBXW7*, and *BRAF* mutations [87]. About 31% of small polyps display two or more pathogenic mutations, with variable allelic frequencies, a finding supporting the presence of multiple tumor cell populations [87].

The large majority of driver mutations are clonal and arise before the start of tumor expansion, thus explaining the existence of only a minimal driver gene heterogeneity among untreated CRC metastases [88]. The rarity of subclonal driver mutations supports the view that subclones may differ by the selective presence of passenger mutations progressively accumulating during growth: these subclones have similar fitness and occupy different tumor regions, thus generating ITH and their size is mainly dependent on the timing of their generation during the process of tumor evolution [89].

Additional evidence in favor of the “Big Bang” model tumor growth comes from additional recent studies. Thus, Williams et al [90] have explored whether the subclonal mutant allele frequencies of a part of cancers of different origin follow a model of tumor evolution based on simple power-law distribution, as predicted by neutral growth. This analysis provided evidence that other cancers, such as stomach, bladder, lung and cervical cancers, as well as CRCs, follow a model of tumor evolution bye neutral growth [90]. In these malignancies, after an initial single tumor expansion, characterized by the formation of multiple heterogeneous subclones that, in spite their genetic heterogeneity, initially grow at comparable rates, without overtaking one another; thus, in these tumors, all clonal selection events occur at a very early stage of tumor development and not in late-developing subclones, thus resulting in the generation of numerous passenger mutations, involved in the generation of intra-tumor heterogeneity [90].

It is commonly believed that passenger mutations have no role in cancer development. However, many passenger mutations fall within protein-coding genes and, although individually weak, these mutations tend to accumulate during tumor progression evading negative selection mechanisms, and in their collective burden, alter the course of tumor progression [91,92].

Lineage tracing experiments in human colorectal adenomas further support the “Bing Bang” theory of colony cancer development [93]. These experiments led to the identification of multipotential stem cells within human colorectal adenomas, responsible for the development and maintenance of these tumors. The study of methylation patterns of non-expressed genes, as well as the analysis of genetic lesions in micro dissected individual crypts from colonic adenomas were used to characterize clonal evolution of these tumors [93]. The analysis of individual crypts within each adenoma showed that adenomatous crypts are clonal populations maintained by multipotential stem cells; individual crypts from each adenoma display different methylation patterns; intratumor clones present in some colonic adenomas are epigenetically homogeneous [93]. The results of this study were compatible with a model of colorectal adenoma evolution not based on continual steady growth but on an initial burst of tumor growth, followed by relative quiescence; the tumor clones form at the initial stages of tumor development but not sweep through the tumor and are present as localized with divergent intraclone methylation patterns. Rare subclones are generated later during tumor development, exhibit homogeneous methylation patterns, and are localized at the level of focal regions of the tumor [93].

Studies of the spatial distribution of genetic alterations within a tumor by phylogeography, an approach that combines tumor phylogeny or the ancestral relationships of tumor subclones with their spatial physical locations in the tumor, allows to visualize how tumors spread [94]. The spatial analysis of private mutations in early CRCs, combining multiregional sequencing with mathematical multiscale models showed the existence of spatial mutation patterns in these tumors, supporting the existence of early colorectal tumor cell mobility, a tumor cell property required for generating ITH [95].

The analysis of epigenetic ITH into CRCs analyzing opposite tumor sides showed evidence of little ITH or stepwise selection during tumor development, suggesting that the epigenome observed in various tumor regions reflects that of its founder cells; despite epigenomic conservation, RNA expression displayed significant variation between individual tumor regions, seemingly due to mechanisms of continue adaptation related to phenotypic plasticity [96].

Saturation microdissection and targeted deep resequencing have shown that CRCs are jigsaw arrayed in millimeter-wide columns sharing common phenotypes rather than being arranged horizontally by phenotype [97]. Most of the large subclones thus identified shared both invasive and superficial phenotype; subclones with invasive phenotypes arose from both early and late phylogenetic branches [97]. This pattern of phylogeography is consistent with single tumor expansions by founder cells possessing all the driver mutations required to sustain tumor growth rather than a stepwise mechanism involving progressive invasions by a minority of subclones at various levels of progression [97]. Particularly, on 11 CRCs analyzed in this study, two out of 11 displayed private driver mutations, while nine in 11 did not have private driver mutations, showing evidence of multiclonal invasion, and invasive and metastatic subclones originate early during tumor development [97].

In conclusion, the analysis of the genetic heterogeneity observed at the level of CRCs is compatible with a “Big Bang” expansion model, characterized by an early phase of tumor growth consisting in a single cell expansion; this initial tumor expansion generates a large number of early-arising clones, coexisting within the tumor for long periods of time for the absence of a selective pressure [98]. This weak selection was insufficient to determine large clonal expansions in short times. This finding supports the view that the large part of tumor heterogeneity is generated early during tumor development, at a stage where the tumor is still undetectable at clinical level [98].

## 9. Conclusions

About half of CRCs develop metastases and metastatic spreading is the main cause of CRC-related death. The dynamics and the molecular processes remain largely unknown. Several recent studies have shown that systemic spread can occur early in CRC development. Recent studies have reported a detailed analysis of the genomic landscape of metastatic CRC patients underlying the molecular heterogeneity of these patients and the possibility to identify some therapeutic targets in these patients. The study of molecular evolution of CRCs suggest that these tumors may evolve either through a process of subclonal selection or neutral evolution.

A better understanding of the cellular and molecular processes governing CRC metastasis spreading will be necessary to improve the outcome of metastatic CRC patients.

Although the survival rate of patients with metastatic CRC patients improved in the last years, the response to current treatments and prognosis of patients bearing *KRAS*, *NRAS*, and *BRAF* mutations remain still poor. Therefore, there is an absolute need to identify these patients and to discover new improvements for therapeutic vulnerabilities and to formulate rational prospective personalized therapies aiming to improve their survival chances.

## Figures and Tables

**Table 1:** The gene expression-based consensus molecular classification of colorectal cancer.

Tumor Subtype	Frequency	Gene Expression	Genetic Abnormalities	Tumor Location	Prognosis
		Signature			
CMS1 Hypermutated	14%	Immune infiltration and activation High PD-1 activation Low stromal cell infiltration	SCNA low Hypermutated MSI high CIMP high KRAS (25%) BRAF (40%) APC (35%) TP53 (30%)	Predominantly proximal (74%)	Intermediate Poor prognosis after relapse
CMS2 Canonical	40%	WNT and MYC activation Elevated expression of EGFR, HER2, IGF2, IRS2 and HNF4A Low immune infiltration and activation	SCNA high No hypermutated MSI low CIMP negative KRAS (30%) BRAF (0%) APC (80%) TP53 (70%)	Predominantly distal (80%)	Good
CMS3 Metabolic	10%	Metabolic deregulation, with upregulation of several metabolic signatures (glutaminolysis and lipidogenesis) Low immune and stromal cell infiltration.	SCNA mixed Hypermutated (30%) MSI low; MSI high (15%) CIMP mixed KRAS (70%) BRAF (10%) APC (75%) TP53 (30%)	Equally proximal and distal	Intermediate
CMS4 Mesenchymal	25%	Stromal infiltration TGF-β activation Angiogenesis Matrix-remodelling pathways Complement-mediated inflammation	SCNA high No hypermutated MSI low CIMP negative KRAS (40%) BRAF (5%) APC (65%) TP53 (55%)	Mainly distal (66%)	Negative Usually diagnosed at advanced stage

SCNA: somatic copy-nucleotide alteration.
